# Dinosaur palaeoneurology: an evolving science

**DOI:** 10.1098/rsbl.2024.0472

**Published:** 2024-12-18

**Authors:** Amy M. Balanoff

**Affiliations:** ^1^ Center for Functional Anatomy and Evolution, Johns Hopkins University School of Medicine, Baltimore, MD, USA

**Keywords:** endocasts, Dinosauria, neuroanatomy, evolutionary neuroscience

## Abstract

Our fascination with dinosaur brains and their capabilities essentially began with the first dinosaur discovery. The history of this study is a useful reflection of palaeoneurology as a whole and its relationship to a more inclusive evolutionary neuroscience. I argue that this relationship is imbued with high heuristic potential, but one whose realization requires overcoming certain constraints. These constraints include the need for a stable phylogenetic framework, methods for efficient and precise endocast construction, and fossil researchers who are steeped in a neuroscience perspective. The progress that has already been made in these areas sets the stage for a more mature palaeoneurology—not only one capable of being informed by neuroscience discoveries but one that drives such discoveries. I draw from work on the size, shape, behavioural correlates and developmental role of the dinosaur brain to outline current advances in dinosaur palaeoneurology. My examples largely are taken from theropods and centre on questions related to the origin of birds and their unique locomotory capabilities. The hope, however, is that these exemplify the potential for study in other dinosaur groups, and for utilizing the dinosaur–bird lineage as a parallel model on a par with mammals for studying encephalization.

## 1. Origin of dinosaur palaeoneurology

The study of the dinosaur central nervous and special sensory systems (palaeoneurology) finds its origin in the same Victorian setting as dinosaurian palaeontology *sensu lato*, just before the early twentieth century origin of modern neuroscience [1]. It was less than 50 years after William Buckland described the first dinosaur bone [2] and fewer than 30 years after Richard Owen coined the name Dinosauria [3] that J. W. Hulke provided the initial description of a dinosaur endocranial cavity—that of the iguanodontid *Mantellisaurus* (NHMUK R2501) [4–6]. Hulke offered this braincase to T. H. Huxley for study, but Huxley’s ‘many and increasing engagements’ [4, p. 200] prevented his participation. It is a fascinating ‘what if’ scenario to think of Huxley, one of history’s great comparative anatomists, applying the full force of his intellect to this endeavour. Huxley was, after all, in the midst of a flurry of publications elucidating the similarities between birds and dinosaurs [7–10]. His predisposal to recognize the avian-like features of the ornithischian endocranial space (see [6,11]) is evidenced by his oral response to Hulke’s presentation at the Royal Society, congratulating him on ‘the progress being made in our knowledge of this interesting group of Reptiles and of their ornithic affinities’ [4, p. 206]. Thus, in the early 1870s Huxley was poised to champion a modern view of the dinosaurian brain, perhaps paralleling his neuroanatomical observations between human and chimp in clarifying our own branch of the primate tree [12] (see [13]). As it was, the initial 1880 description of dinosaur endocasts—the infilling of the endocranial space providing an estimate of brain morphology—was provided by Yale palaeontologist O. C. Marsh who noted the ‘lacertilian rather than avian’ nature of *Morosaurus* (*Smitanosaurus*; *sensu* [14]) and *Stegosaurus* [15, p. 254]. The ‘lizard-like’ filter that influenced Marsh’s thinking continued to dominate dinosaur palaeontology for the next century.

The long, historical relationship between palaeoneurology (see [16] for historical timeline) and modern neuroscience, defined by the work of Ramon y Cajal (see [17]), may be accurately described as unidirectional—with information and influence flowing from latter to former. Denial of a healthier relationship of reciprocal illumination was driven by one obvious and unchangeable reality and three historical (but mutable) constraints. The unchangeable reality is that the empirical basis of palaeoneurology is not neural tissue itself but its interaction with the neighbouring skeleton. This fact certainly limits the range of neuroscience questions that palaeoneurology can address. But limitation does not equal negation, and there is unfulfilled potential that may soon permit palaeontology a seat at the high table of evolutionary neuroscience. Occupying this seat requires us to recognize and overcome the limitations of the past. I see these constraints as (i) lack of a clear phylogenetic framework, (ii) restrictions on systematic sampling and (iii) disciplinary disparity. I will begin by outlining each historical constraint and making an argument for how they are being addressed in dinosaur research. I will conclude by summarizing our current, collective progress in dinosaur palaeoneurology and predict what questions are likely to drive the next stage of research.

## 2. Historical obstacles to an integrated palaeoneuroscience of dinosaurs

### (a) Phylogenetic framework

It is hardly a novel insight that the lack of a well-supported phylogenetic framework served as a long-standing obstacle to evolutionary studies generally and palaeontological studies specifically. In the absence of this framework, the role of fossils in establishing the origins of extant groups and their biology remained murky, naturally eroding the influence of palaeontology in a modern, synthetic biology. This lack of a clear heuristic role certainly applied to fossil endocasts and helps explain why the pre-phylogenetic history of their study largely failed to mature beyond ad hoc description. For dinosaurs, the cladistic analyses of Jacques Gauthier [18,19] established a modern framework for the relationships among the extinct dinosaur lineages and the paraphyletic status of these lineages with respect to birds. Given that (i) all fossils lie along some phylogenetic lineage and (ii) diagnostic features of a crown clade (a group defined by its living forms) evolved along its phylogenetic stem, a primary role of fossils in biological studies is to inform the timing and context in which these crown features accrued—something that cannot be done by studying extant species alone.

The importance of fossils in this role scales positively with the length of the involved stem. Despite rate variations in morphological evolution, relatively long stem lineages tend to circumscribe higher numbers of transformations, many of which may be far removed from their modern biological context defined by the crown. The avian stem lineage, connecting the last common ancestor of birds and crocodylians with that of all living birds, is at least 110 Myr long [20,21]. Such length means that endocasts of non-avian dinosaurs (which dominate this stem lineage) are essential for understanding the origins of the highly encephalized, highly derived behaviour of living birds. For example, the crocodile–bird phylogenetic bracket indicates that the expanded, nucleated cerebral cortex of living birds originated from a plesiomorphic three-layered cortex [22]. The details of when, where and potentially how this arrangement first appeared along the avian stem are important given the huge implications that cerebral expansion and reorganization are thought to have for bird cognition and behaviour [23,24]. This history can only be revealed if we establish what details endocasts can and cannot provide and then analyse these data in a robust, phylogenetically informed manner. Recent phylogenetic work, driven by cosmopolitan field discoveries (e.g. [25,26]), has produced a detailed stage on which still-emerging comparative methodologies may establish patterns of neuroanatomical transformation. The issue is whether there are enough data to support meaningful results.

### (b) Sampling restrictions

**Figure 1.**
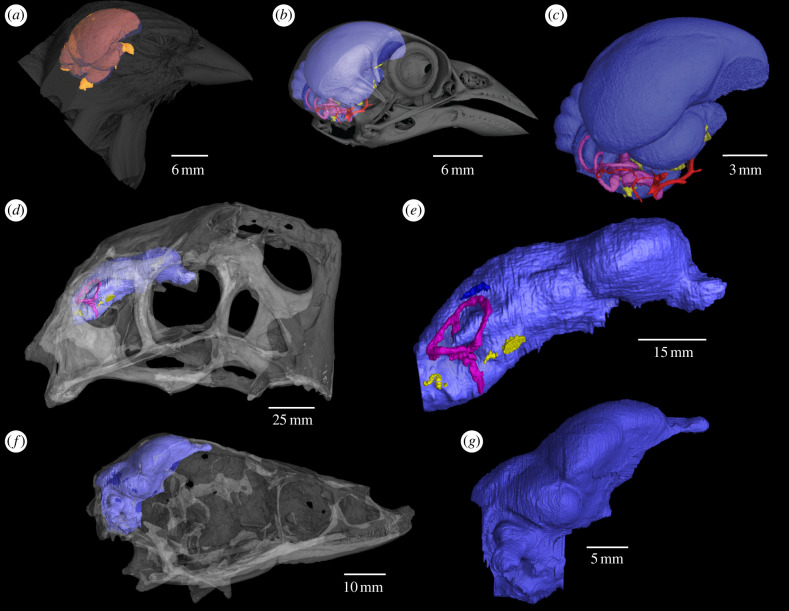
Digital renderings of endocranial structures. (*a*) Relationship of the brain (orange) to the endocast (blue) within the transparent head of sparrow, *Passer domesticus*. (*b*) Endocast, endosseous ear labyrinth (pink) and surrounding nerves (yellow) and vasculature (red) in *P. domesticus* with skull rendered transparent. (*c*) Endocast and surrounding structures in *P. domesticus*. (*d*) Endocast, endosseous labyrinth and cranial nerves in the oviraptorid theropod *Citipati osmolskae* (IGM 100/978) with skull rendered transparent. (*e*) Endocast and surrounding structures of *C. osmolskae*. (*f*) Endocast and transparent skull of unnamed troodontid theropod IGM 100/1126. (*g*) Endocast of the same unnamed troodontid.

Palaeoneurology relies heavily on endocasts (figure 1). The source of these endocasts long was restricted to either highly fortuitous preservation, or rather crude, destructive methods (e.g. [15,27]). Natural endocasts, representing sedimentary infillings of the cranial cavity, require a Goldilocks level of erosion—enough to permit exposure, diagnosis and study but not so much that features are lost. Artificially produced casts (e.g. [28]) wrest some control from taphonomy but usually require physically opening the endocranial space for preparation—far from ideal when working with treasured, *n* = 1, specimens.

A true turning point is represented in the efforts of Timothy Rowe, who in the 1990s helped create a laboratory at the University of Texas at Austin, whose purpose, in part, was (and is) to produce high-resolution computed tomographic (CT) data of fossils [29]. This non-destructive method bears the inherent potential to produce and study anatomically detailed, digital endocasts with relative ease [30–33]. Today, most centres of palaeontological research include a CT laboratory, with endocast construction being standard operating procedure. Digital approaches, now including X-ray synchrotron [34] and neutron X-ray CT [35], also facilitate the objective retrodeformation of taphonomic damage [36]. This expansion of sampling potential will likely prove critical in studying the neuroanatomy of a long-elusive portion of avian deep history lying between *Archaeopteryx* and the crown. Known avialan endocasts include *Archaeopteryx*, *Ichthyornis*, *Hesperornis* and *Cerabavis* [37–41] and the recently described *Navaornis* [42], but a large majority of the dense avialan record is represented by slab specimens whose endocasts are becoming increasingly accessible. A scenario where every adequately preserved dinosaur braincase is sampled for an endocast, with those endocast data incorporated into broad-based, phylogenetically informed, evolutionary analyses, is not only realistic but perhaps imminent. It seems clear that these efforts will soon be aided by machine learning algorithms capable of highly precise retrodeformation and segmentation.

Digital endocasts have the added advantage that they are inherently easy to share. As the community of researchers and educators with access to these data increases, so will the number, quality and influence of the resultant studies. Efforts to maximize the logistical efficiency of archiving, searching and distributing these data will no doubt continue [43]. MorphoSource.org stands as the current flag bearer in these efforts, but like all such endeavours will need to balance the shifting needs of its community of users with the inherently tenuous struggle to maintain adequate funding.

A digitized endocranium eases integration of stem and crown neuroanatomical data in other ways (e.g. [31,39–41,43–51]). CT is equally applicable to extant taxa, and when extended to related modalities, such as diceCT [52], can better establish the anatomical origins of endocast features [51]. Endocast-informed studies of brain evolution must consider the degree to which endocast morphology conforms to brain morphology, as opposed to other cranial features—e.g. endocranial nerves, vasculature [53], and dural sinuses [54], or even extracranial adductor muscles [55]. The Brain-to-Endocranial Cavity (BEC) index [56,57] is a measure of an endocast’s power to predict the surface anatomy of the brain and thus its potential to inform neuroanatomical questions (figure 1). Conformation between brain and endocast will never be completely uniform within a single endocranial cavity, and BEC should be expected to vary ontogenetically and phylogenetically [48,58,59]. The latter is well evidenced in bird-line archosaurs where the high BEC index of the crown does not extend along the entirety of the stem. Ornithischian dinosaurs tend to retain large occipital sinuses [11] that at least partially obscure underlying regions and metrics of interest such as the inner ear labyrinth [60–62], floccular fossa [60,63,64] and relative cerebral size [64,65].

The timing and tempo of BEC expansion along the phylogenetic backbone of birds is a topic of ongoing analysis and debate in dinosaur palaeoneurology. The recent study of Herculano-Houzel [66], for example, used endocast size to infer telencephalic neuron numbers in non-avian dinosaurs. Resultant estimates placed *Tyrannosaurus rex* within the range of some primates, implying their cognitive abilities were on a par with modern birds, like crows, that manufacture and use tools [66]. My point here is to neither critique nor promote these findings (see [67,68] for expanded treatment) but simply to point out that such inferences are heavily reliant on our understanding of the BEC index and its relationship to other neuroanatomical and cognitive features.

The sampling benefits of CT also extend to special sensory organs. The inner ear probably has received the majority of research attention, reflecting both the degree to which it is circumscribed within the ossified otic capsule and the range of expressed variation (see below). CT-based studies of dinosaur optic- and olfactory-related properties also exist (e.g. [69–72]), but with much work left to do. The important point here is that we are well on our way to breaking many of the constraints that have limited our ability to sample palaeoneurological data with precision and efficiency. With this release comes the empirical opportunity for palaeoneurology to mature as a vibrant, semi-independent discipline capable of supporting its own community of experts.

### (c) Disciplinary disparity

The perhaps unsurprising reality is that palaeoneurology has been, and largely still is, the purview of palaeontologists rather than neuroscientists. This emphasis of training means that most endocast descriptions are made by those who perceive endocast morphology within a rich context of geologic, phylogenetic and skeletal history but with a relatively sparse understanding of neuroanatomical and neurophysiological properties. As such, endocasts tend to be discussed more as abstracted shapes (albeit using neuroanatomical nomenclature) than as the product of structurally complex neural tissue with equally complex functionality. This situation will always result in an unfulfilled potential for endocasts to inform neuroscientific questions. I am not critiquing palaeontology, which is already a multidisciplinary exercise, and not even the highest functioning palaeontologist can acquire specialized training in every relevant field. But in order for palaeoneurology to realize its potential, its leadership requires the benefits of adequate training.

This does not imply that the history of palaeoneurology is devoid of such figures. Its early period, for example, benefited from the interests and efforts of Ottilie ‘Tilly’ Edinger (1897−1967), who trained as a neuroscientist at the University of Frankfurt and whose father also was a famous neuroscientist (Ludwig Edinger, 1855−1918). After beginning her career at the Geologisch-Paläontologisches Institut and the Senckenberg Museum in Frankfurt, the Jewish Edinger fled Nazi Germany in 1939, and with the support of Alfred Romer (1894−1973) spent the remainder of her career at Harvard [73–75]. Edinger was a prolific researcher and coined the term ‘palaeoneurology’. She not only catalogued and critiqued the palaeoneurological work that preceded her [76,77] but produced prodigious amounts of original data that are still commonly cited (see [73,74]). Edinger viewed endocasts through the eye of a neuroscientist and in so doing explicitly attempted to enrich the comparative neuroanatomy of living taxa with the unique perspective that deep time affords.

A second figure that must be noted here is Harry Jerison (died 2023), who trained in psychology at the University of Chicago. Jerison brought palaeoneurology into a more analytical phase by incorporating fossils into his statistical and theoretical studies on brain size and ‘intelligence’—proposing the encephalization quotient (EQ), a measure of relative brain size, as a means of comparison [78]. Although EQ is out of favour as a metric of intelligence [56,79], his estimates for primarily non-theropod dinosaurs [80–82] helped dispel the extreme dim-witted status afforded to these groups by early dinosaur workers such as Marsh, who were influenced by the allometric notion that extreme increases in body size would result in relative brain sizes among dinosaurs that were well below those of modern lizards and crocodylians. By demonstrating that dinosaur EQs actually fell *within* the range of variation of living non-avian reptiles, Jerison set the stage for reinterpreting their behavioural ecology once a new phylogenetic framework was in place and endocasts from the more bird-like, more encephalized theropods became available. This subsequent revision of dinosaur brain size started with Hopson [83,84] and continues to the present.

Here we again find opportunity for expanding the scientific potential of palaeoneurology, this time through the development of current and future researchers who, like Edinger and Jerison, are steeped in a neuroscience perspective. The efforts and influence of these two researchers also serve as evidence that the palaeoneurology of dinosaurs will likely remain at the forefront of this collective effort.

## 3. Present and future of dinosaur neuroscience

### (a) Endocast size

A general absence of molecular data in the fossil record means that the internal tree topology of a stem lineage will likely always be less stable than that of a well-studied crown clade (see [85]). Still, stem lineages that combine high levels of research interest with high preservation potential will eventually coalesce around a stable topology. For the non-avian lineages of Dinosauria, field-based discoveries and novel descriptions aided by advanced imaging retain the power to reveal unique character combinations that shift tree support and thus phylogenetic hypotheses. It is also true that such insights are increasingly valued for their influence on patterns of structural and functional change inferred across a reasonably stable tree topology. This certainly describes the current impetus for constructing and analysing digital endocasts. An increasingly stable tree, dense sampling of both crown and stem endocasts, and sophisticated comparative methods [86]—an increasing list including reconstruction of ancestral states [87–89] while employing various models of evolution [87,90] and comparing multiple phylogenetic hypotheses [91], character correlation analyses [92], testing for multiple adaptive [93] and functional [70,94–96] scenarios, estimating evolutionary rates and divergence times [97–99]—sets the evolutionary stage for a modern palaeoneurology of dinosaurs.

**Figure 2.**
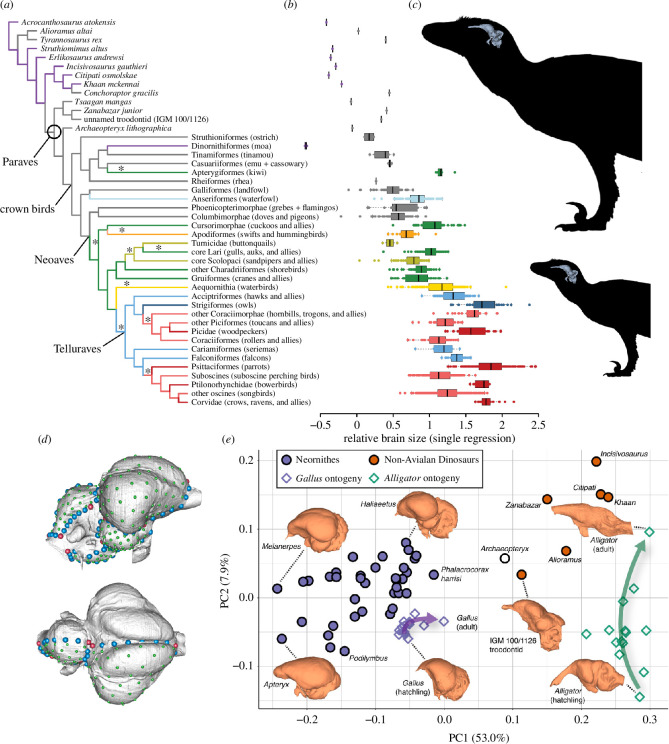
Phylogenetic comparative methods for brain size and shape in coelurosaur theropods. (*a*) Phylogenetic tree of coelurosaurs based on [101,102]. Changes in colour represent significant shifts in relative brain size. Asterisks represent significant changes in the allometric relationship between these variables. (*b*) Residuals of these relative brain sizes plotted as a single regression. (*c*) Outlines of early paravian with brains illustrating how the decrease in brain and body size results in an overall larger relative brain size. (*d*) Digital endocasts of chicken with geometric morphometric landmarks. (*e*) Morphospace of first two principal components of endocast shape. (*a,b*) Modified from Ksepka *et al*. [21]. (*d*) Used with permission from Forcellati *et al*. [103]. (*e*) Used with permission from Watanabe *et al*. [49]. Silhouettes by Emily Willoughby and available on phylopic.org.

A recent demonstration of this analytical potential is Ksepka *et al*. [21], which analysed the volumetric sizes of endocasts from 12 stem- and over 2000 crown-group species. The addressed questions included (i) what are the major patterns of relative brain size evolution in crown and stem theropods, (ii) what was the timing and tempo of this evolutionary change and (iii) what adaptive scenarios best explain the inferred evolutionary patterns? Results generally support the hypothesis of Hopson [83] and Larsson *et al*. [100] that encephalization began early, and the last common ancestor of birds and troodontids (Paraves) was highly encephalized—at the same level as the ancestral crown bird (figure 2*a*,*b*). The Ksepka *et al*. [21] study also showed that this pattern of encephalization was not necessarily driven by positive selection for a large brain and expanded intelligence, which was the seemingly reasonable position. By treating brain and body size individually, the analyses showed that *both* brain and body size were decreasing at Paraves, with encephalization the product of body size decreasing at a faster rate (figure 2*c*). It might thus make better sense to identify smaller body size as the primary focus of any positive selection, perhaps as part of a shift from a terrestrial to more arboreal existence. The rate at which body and brain sizes were evolving among early paravians was higher than inferred rates within the crown, suggesting that an early period of experimentation was supplanted by a more constrained evolutionary dynamic that may reflect the biomechanical demands of flight. The Ksepka *et al*. [21] study thus demonstrates the possibilities of bringing patterns of endocast size into a more mature evolutionary scenario.

### (b) Endocast shape

No one denies that size is an incomplete measure of a brain’s functional potential. Brain size as a whole or of specific neuroanatomical regions could remain static while underlying architecture evolves in functionally significant ways. For example, a brain region that folds increases its surface area and potential neuron number without necessarily expanding its occupied volume. Such a scenario appears to describe the avian cerebellum that, unlike the homologous region of other living reptile groups, is highly folded. The exact position along the avian stem where folding first evolved is unclear. There is, however, an apparent volumetric expansion of the cerebellum at the base of Maniraptora [40,41,104]. Patterns within the crown are complicated by folding with relative volumes being larger and smaller than their stem relatives [104]. Even when considering the potential influence of dural sinuses (sometimes reflected on the endocast resulting in overestimation of volume), this is a paradoxical signal that almost assuredly reflects the limitations of volumetric comparisons and should serve as a reminder that volumetric data are best interpreted in the context of other sources of variation such as shape [105].

Recent geometric morphometric (GM) analyses utilized increasingly dense digital landmarks and semi-landmarks to capture the shape of the avian skull and endocast [49,50,106,107]. The inherent difficulty of identifying an adequate number of homologous landmarks on a globular structure like the brain may ultimately be overcome with landmark-free analyses such as elliptical Fourier or spherical harmonics [108]. Recent studies of non-avian dinosaurs suggest incongruent patterns of shape and volumetric transformation in the deep history of birds [50,109–112]. The three-dimensional GM study of Watanabe *et al*. [50] explicitly found that the stem history of endocast shape diverges substantially from its volumetric history (figure 2*d*,*e*). This disparity includes finding that the critical volumetric expansion recovered at the base of Paraves [21] was not accompanied by a significant transformation in shape. The Watanabe *et al*. [50] study also found that the avian crown, which does not have an especially unique volumetric signature, does have an autapomorphic shape. The functional implications of this size–shape disparity remain unclear, but that the disparity exists is an argument against using either of these metrics in isolation.

For the example of avian cerebellar folding, shape analyses have yet to be combined with volumes to trace its origin among non-avian theropods. However, such a study represents a woefully underexplored form of integrated shape analysis. As noted above, even with an ideal BEC index of 1, an endocast will reflect only the surface features of the associated brain. It is thus incumbent on palaeoneurology to determine how the size and shape of the brain’s surface morphology correlate with its functionally significant, internal architecture. Given the complexity of the brain as a whole, these analyses need to be carried out in specific regions, and the more exclusive the better (e.g. [51]). The covariance of these properties will be another important measure of an endocast’s neuroanatomical potential.

### (c) Predicting function/behaviour

The dinosaur literature teems with studies using overall endocast size to draw conclusions regarding the functional and/or behavioural characteristics of individual species. The often-unacknowledged theoretical basis for such assessments is the principle of proper mass (PPM) [82]. PPM states that the relative size of a neuroanatomical region predicts its functional importance, as reflected in the number of associated neurons, which in turn reflects the region’s/structure’s overall size. Even setting aside the aforementioned problems that dynamics like folding have for precise employment of PPM, its application is often extended to the complex and vaguely defined functions of intelligence/cognitive ability. Firmer PPM footing may be found in sensory regions, which tend to support fewer functions overall and thus have clearer relationships to the components of a complex behavioural repertoire. Again, the idea is that the relative size of a sensory region reflects the proportion of dedicated neurons and thus the importance of that sensory input for the biology of the organism [113].

A classic application in dinosaurs is olfactory bulb size, with disproportionally large bulbs indicating a disproportionate influence of olfactory processing on behaviour. This correlation was used to infer olfactory acuity across many dinosaurian groups [69,114,115], supporting conclusions such as the ability of tyrannosaurs to employ olfaction in scavenging. Other early coelurosaurs, such as ornithomimisaurs and oviraptorosaurs, were much more visually oriented with small olfactory bulbs suggestive of the modern bird condition [69,115]. Similarly, perceptible auditory frequencies can be reconstructed based on cochlear canal length [116]. Multimodal sensory regions, such as the optic tecta where visual cues are integrated with other sensory information, can estimate their behavioural role so long as one type of sensory input dominates. This caveat is drawn from studies of nocturnal animals, like the kiwi, that have small optic tecta and are heavily reliant on non-visual sensory information [117].

The shape of sensory structures may also be useful behavioural predictors. As noted above, much of this research in dinosaurs has been directed toward the inner ear, especially the semicircular canals. These canals imperfectly circumscribe the membranous semicircular ducts, which house the specialized epithelium of balance and spatial orientation. These sensory structures drive the vestibulo-ocular and vestibulo-collic responses and thus control compensatory adjustments of the eyes and neck, respectively, as the head and body move during angular acceleration [118]. It is thus reasonable to predict that canal morphology reflects, at least in part, behavioural properties related to movement [119,120]. A pair of recent studies targeting the more inclusive archosaur radiation but that sample densely within crown and stem dinosaurs represent well the promise of the comparative approach and the fact there is much we need to learn. The study of Hanson *et al*. [121] is a comparative shape analysis of the entire inner ear endocast, inclusive of the semicircular canals, vestibule and cochlea, with observed variation aligning well with locomotor styles partitioned as quadrupedal, bipedal and low-manoeuvrability fliers, and high-manoeuvrability fliers. The nearly concurrent study of Bronzati *et al*. [122] drew somewhat contrasting conclusions in finding that canal shape correlates more closely with skull shape than with locomotory mode—in agreement with an earlier analysis of flight style in living birds [123]. Well-devised developmental and *in vivo* functional experiments may shed light on these correlations thereby representing another underutilized form of reciprocal illumination in palaeoneurology—that which exists between pattern- and experimental-based inference.

**Figure 3.**
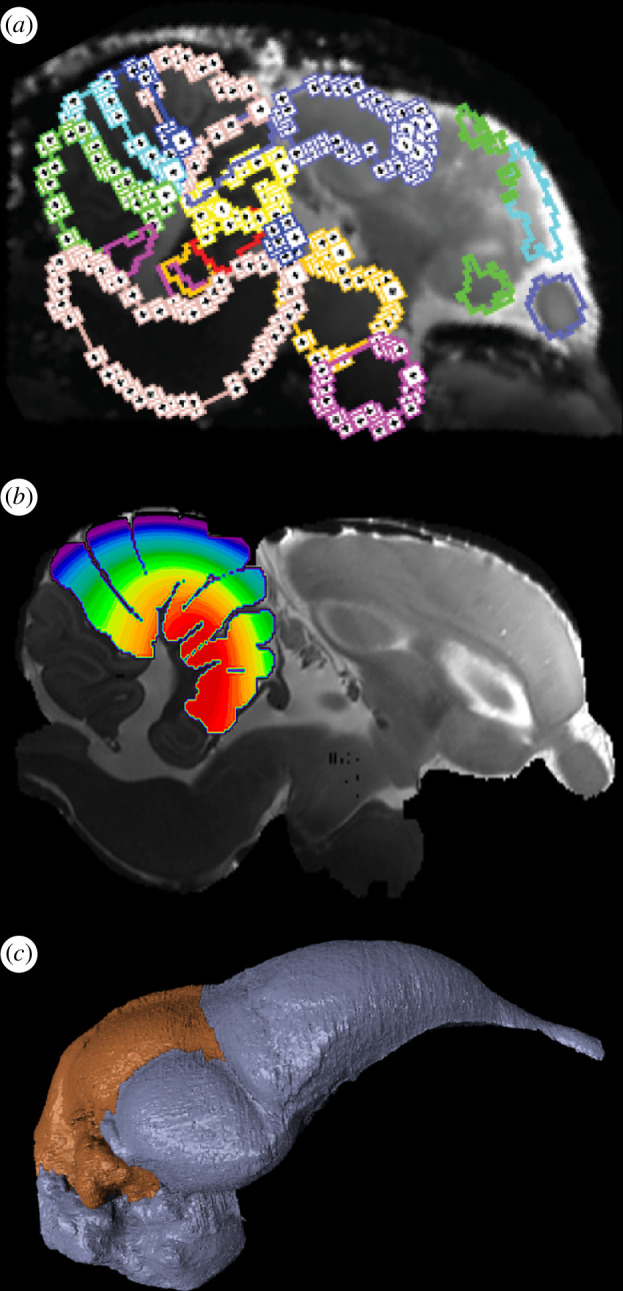
Neurophysiological data on brain activity can be integrated into a larger examination of neuroanatomy and deep evolutionary history of behaviour. (*a*) Magnetic resonance imaging data of a pigeon brain with neuroanantomical partitions segmented that is co-registered to (*b*) positron emission tomography data displayed as a heat map immediately after flight. (*c*) Endocast of the avialan *Archaeopteryx lithographica* with the cerebellum isolated (orange). After finding significant increases in activity in the cerebellum during flight, regional volumetric and shape data can be used to reconstruct the evolutionary history of this structure relative to other anatomical indicators of flight. (*a*,*b*) Modified from [104].

A rare example of experiments designed specifically to inform, and be informed by, observed patterns of dinosaur palaeoneurology is the recent study of Balanoff *et al*. [104]. The study was initiated to address whether the evolutionary origin of avian flight within non-avian dinosaurs was accompanied by neurological changes observable in the endocast (figure 3). While the time-calibrated fossil record of flight potential, based on post-cranial indicators such as limb proportions and feather structure, may be correlated with size and shape changes in the endocast, meaningful functional interpretations of these patterns require some understanding of whether avian flight is supported by a unique pattern of brain activity and what that pattern entails. Positron emission tomography (PET) was used to quantify whole brain activity in flying pigeons and compare it with the baseline signature of rest (figure 3). Whole brain data are important given that complex behaviours likely involve multiple brain regions or pathways [124]. The dominant activity pattern was one of stasis across behaviours, albeit with certain significant differences. Flight activity was concentrated in the cerebellum, particularly regions processing somatosensory, optic flow and local motion information (figure 3*b*). Among the differentially active optic flow pathways were those controlling the vestibulo-ocular and vestibulo-collic response, indicating that rotational and translational information from the inner ear was active during flight as predicted by Witmer *et al*. [125]. Regions processing local (parallax) motion and proprioception showed an even stronger flight-specific signal perhaps reflecting the complex action of landing. Experimental confirmation that the cerebellum has a disproportionate role during flight was then used to inform patterns of endocast evolution. The potential for powered flight is now considered to have been in place at least by the origin of Paraves [126] and perhaps as early as Pennaraptora [127]. Directly preceding the phylogenetic origins of these dinosaur clades was an expansion of the cerebellar region of the maniraptoran endocast, suggesting that a brain with the cerebellar capability of supporting an advanced locomotory style was in place prior to the origin of flight and may be considered an exaptation for this behaviour.

The Balanoff *et al*. [104] study is simply a first step in understanding the behavioural implications of dinosaur neuroanatomy, but the insights it provides bode well for the future of palaeoneurology as an integration of pattern and experiment. In addition to PET, functional magnetic resonance imaging [128], activity-dependent immediate early genes like *c-fos* [129,130] or electrophysiological EEG readings [131] all may be used to produce behaviour-informed neural activity data that can be co-registered with neuroanatomical atlases to establish a direct line of inference from behaviour to neural activity to neuroanatomy to fossil endocast.

### (d) Evo-Devo and the dinosaur brain

The precocial timing of neurogenesis grants the vertebrate brain a developmental primacy over most other cranial and postcranial tissues. Such primacy means that evolutionary transformations in brain development bear the potential for a wide range of downstream patterning effects and structural change. This generalized potential of the brain in Evo-Devo studies is heightened in dinosaurs given the nearly unique nature of their encephalized condition. The deep history of the avian brain constitutes a model system that lies in parallel with that of primates and bears the associated potential for reciprocal illumination with our own evolutionary past.

Birds with the largest relative brain sizes, like crows, share with hominins [132] a marked allometric shift in the growth relationship between brain and body size [21]. In hominins, this shift is explained by an extended period of cerebral development [133]. Whether the same heterochronic mechanism underlies avian encephalization is unknown. It is of interest that the significant paravian expansion of brain size entails no significant change in brain–body size allometry but rather a shift in *y*-intercept within an otherwise stable pattern of covariation [21]. While this may reflect selection for body size and thus encephalization through miniaturization [134–136], it does not negate similarities with the developmental mechanisms of primate encephalization. There is evidence that the morphology of the brain and surrounding cranial structures undergo a paedomorphic shift at this point on the tree, becoming more ‘juvenile’ and s-shaped in form [109,137] (but see [50]). More importantly, here we see the potential for combining bird and primate data to better understand encephalization as an evolutionary process—e.g. its tendency to utilize homologous developmental pathways, down to and including the identity of involved genes and their regulatory dynamics.

The primacy of the brain also grants it the potential to act as a critical signalling centre during development with a role in patterning the morphogenesis of neighbouring tissues and thus regulating the degree to which these tissues are integrated or behave as semi-independent modules [47,138–141]. When neural and skeletal tissue share a developmental influence (through either one-way signalling or crosstalk), the history of this relationship may be informed by the comparative analysis of endocasts. Fabbri *et al*. [47], for example, used endocasts to promote a formative developmental relationship between the brain and overlying cranial roof. The structural correlation between the forebrain–frontal bone and midbrain–parietal bone across the base of the amniote radiation and into the stem groups of both birds and mammal implies a highly conserved developmental connection that actually informs the long-debated homology of the avian frontal and parietal [142]. The cerebral expansion characterizing the avian and mammalian crown clades either served to break this developmental constraint or represents a product of that breakage.

Perhaps just as interesting is establishing which cranial tissues and structures maintain a developmental and evolutionary independence from neural transformations. Given that highly encephalized crown birds have a relatively small adductor chamber, it makes sense that these systems are developmentally and evolutionary connected, potentially reflecting a trade-off scenario. The discovery that the highly encephalized, near-crown stem bird *Ichthyornis* retains a basically plesiomorphic adductor chamber [39] casts serious doubt on this trade-off hypothesis, prompting Cerio *et al*. [55] to test for developmental correlations between endocast and adductor muscles, which they did not find. Each of these examples reflects the rich opportunities that endocast-informed, Evo-Devo studies of the bird brain have for understanding the mechanistic details and evolutionary implications of encephalization.

## 4. Conclusions

Palaeoneurology as practiced through the study of endocasts has a deep history, but one whose potential for discovery has been dampened by certain empirical, logistical, technological and disciplinary constraints. The fundamental structure of these constraints has now largely been broken, setting the stage for a fuller and more mutually beneficial relationship with an inclusive evolutionary neuroscience. The examples I used to establish these historical points and to illuminate a path for future studies are drawn primarily from one partition of the dinosaur tree—the theropod lineage most closely related to the avian crown. This is no way diminishes the importance of endocast studies in other dinosaur lineages and the need to continue expanding these endocast data beyond the purely descriptive phase of our science. The dinosaur fossil record maintains its ability to inspire research and to inform a wide range of fundamental evolutionary questions. This combination certainly applies to the structure, function, development, and evolution of the dinosaur brain.

## Data Availability

This article has no additional data.
